# Supplementary material for: The small iron-deficiency-induced protein OLIVIA and its relation to the bHLH transcription factor POPEYE
Source: PLoS One. 2024 Apr 16;19(4):e0295732. doi: 10.1371/journal.pone.0295732 (PMC11020826; doi:10.1371/journal.pone.0295732)
Supplement: S1 Fig — Co-expression network of Fe deficiency-responsive genes. OLV belongs to a sub-network of FIT target genes (surrounded by a blue dashed line). FIT is a transcription factor required for up-regulated expression of FIT target genes in response to Fe deficiency in roots of seedlings (Schwarz and Bauer, 2020). PYE belongs to a sub-network of Fe homeostasis genes (surrounded by a red dashed line) up-regulated upon Fe deficiency in roots and shoots of seedlings, also in the absence of FIT (Schwarz and Bauer, 2020). The ATTED-II tool (Ver. 9.2) was used to generate the network (Obayashi et al., 2018) based on PYE and OLV as input genes. The violet arrow links the two genes encoding the interacting proteins PYE and OLV. (PDF) [file pone.0295732.s001.pdf]

S1 Fig

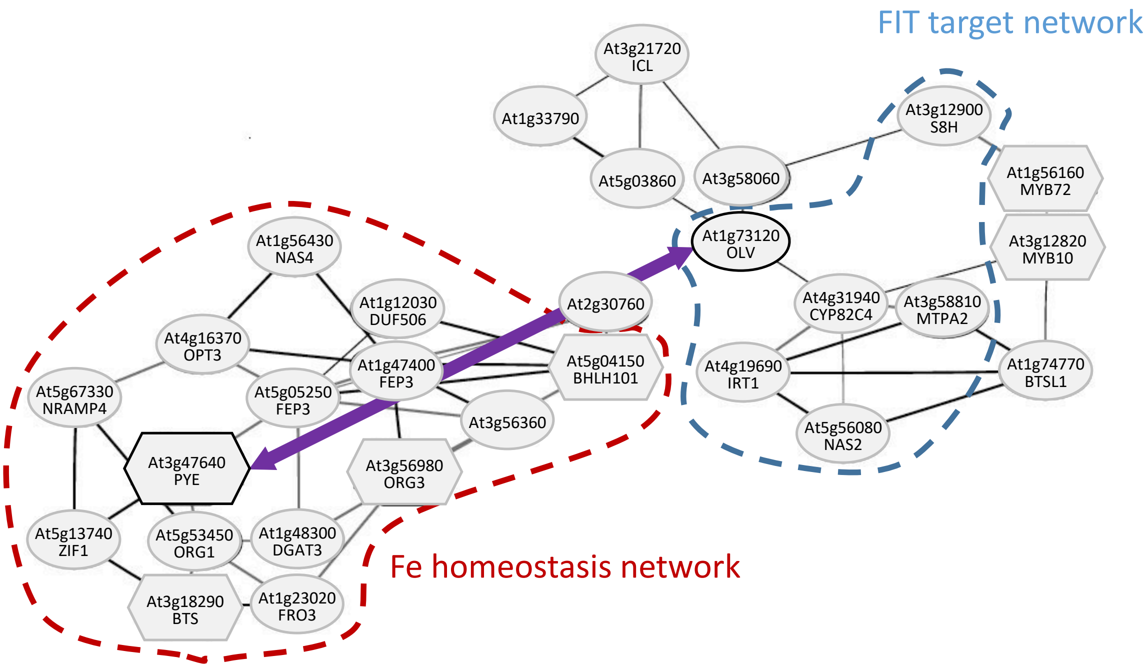

S1 Fig. Co-expression of *PYE* and *OLV* genes

Co-expression network of Fe deficiency-responsive genes. *OLV* belongs to a sub-network of FIT target genes (surrounded by a blue dashed line). FIT is a transcription factor required for up-regulated expression of FIT target genes in response to Fe deficiency in roots of seedlings (Schwarz and Bauer, 2020). *PYE* belongs to a sub-network of Fe homeostasis genes (surrounded by a red dashed line) up-regulated upon Fe deficiency in roots and shoots of seedlings, also in the absence of FIT (Schwarz and Bauer, 2020). The ATTED-II tool (Ver. 9.2) was used to generate the network (Obayashi et al., 2018) based on *PYE* and *OLV* as input genes. The violet arrow links the two genes encoding the interacting proteins PYE and OLV.
