# Supplementary material for: The small iron-deficiency-induced protein OLIVIA and its relation to the bHLH transcription factor POPEYE
Source: PLoS One. 2024 Apr 16;19(4):e0295732. doi: 10.1371/journal.pone.0295732 (PMC11020826; doi:10.1371/journal.pone.0295732)
Supplement: S2 Fig — (A) Multiple sequence alignment of OLV full length protein sequences from angiosperms. The ortholog with the highest maximum similarity score of each order is shown. Arabidopsis thaliana OLV is boxed in red. Conserved amino acid residues are shadowed in color. Below is the consensus sequence, with indicated TGIYY motif. See S3 Fig. (B) Sequence alignment of the OLV TGIYY motifs from A. thaliana and rice, highlighting a consensus. (PDF) [file pone.0295732.s002.pdf]

S2 Fig

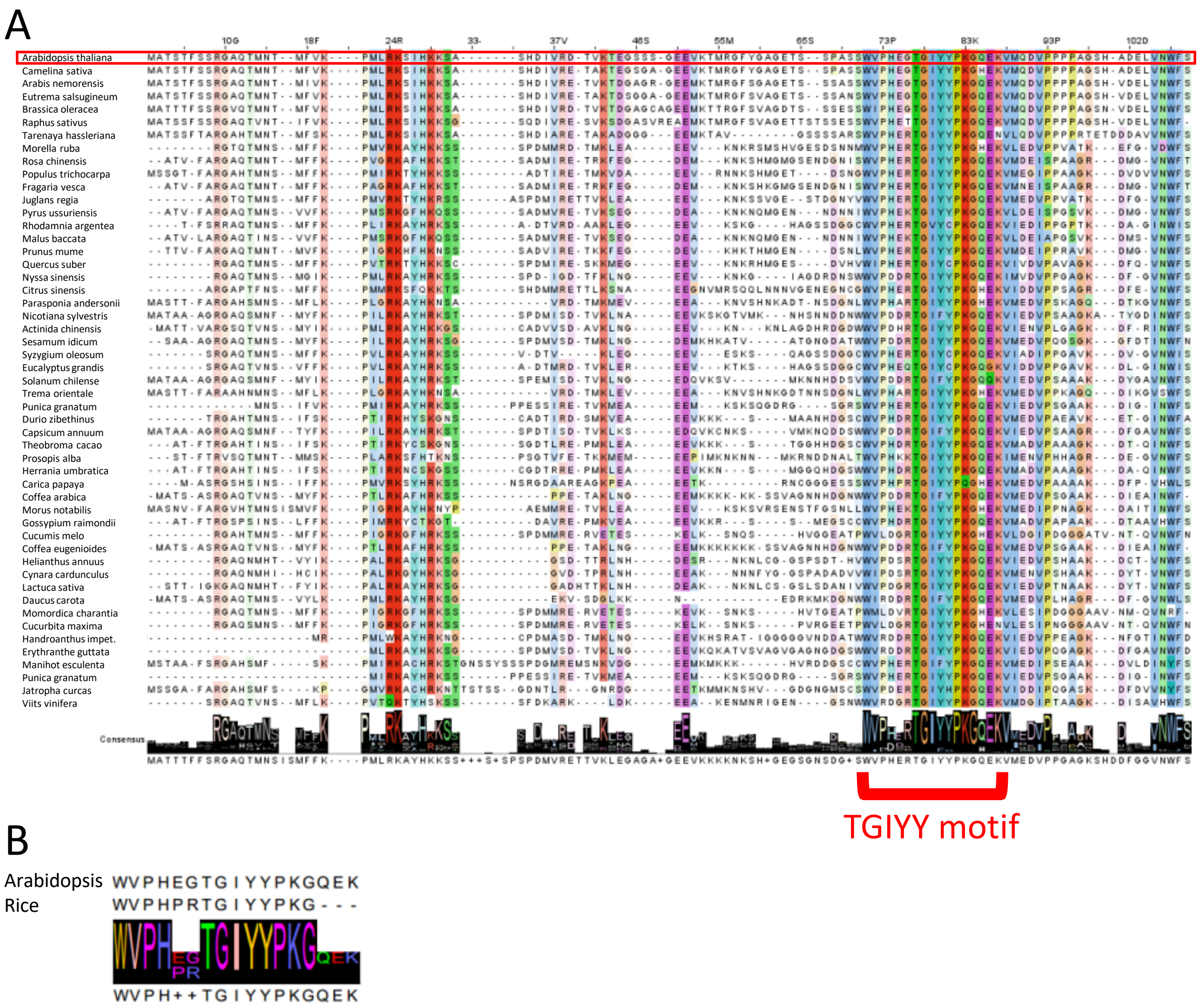

S2 Fig: OLV full-length protein sequence alignments

(A) Multiple sequence alignment of OLV full length protein sequences from angiosperms. The ortholog with the highest maximum similarity score of each order is shown. *Arabidopsis thaliana* OLV is boxed in red. Conserved amino acid residues are shadowed in color. Below is the consensus sequence, with indicated TGIYY motif. See Figure S3. **(B)** Sequence alignment of the OLV TGIYY motifs from *A. thaliana* and rice, highlighting a consensus.
