# Supplementary material for: The small iron-deficiency-induced protein OLIVIA and its relation to the bHLH transcription factor POPEYE
Source: PLoS One. 2024 Apr 16;19(4):e0295732. doi: 10.1371/journal.pone.0295732 (PMC11020826; doi:10.1371/journal.pone.0295732)
Supplement: S3 Fig — (A) Schematic representation of full length OLV and its amino acid sequence with N-terminus in blue, conserved TGIYY motif in red, C-terminus in green. (B) Multiple sequence alignment of the OLV TGIYY motifs of angiosperms. The ortholog with the highest maximum score similarity of each order is given. Multiple sequence alignments and consensus depicting TGIYY motifs from proteins found in (C), selected green algae and non-angiosperm land plants, and (D), non-plant organisms. (PDF) [file pone.0295732.s003.pdf]

S3 Fig

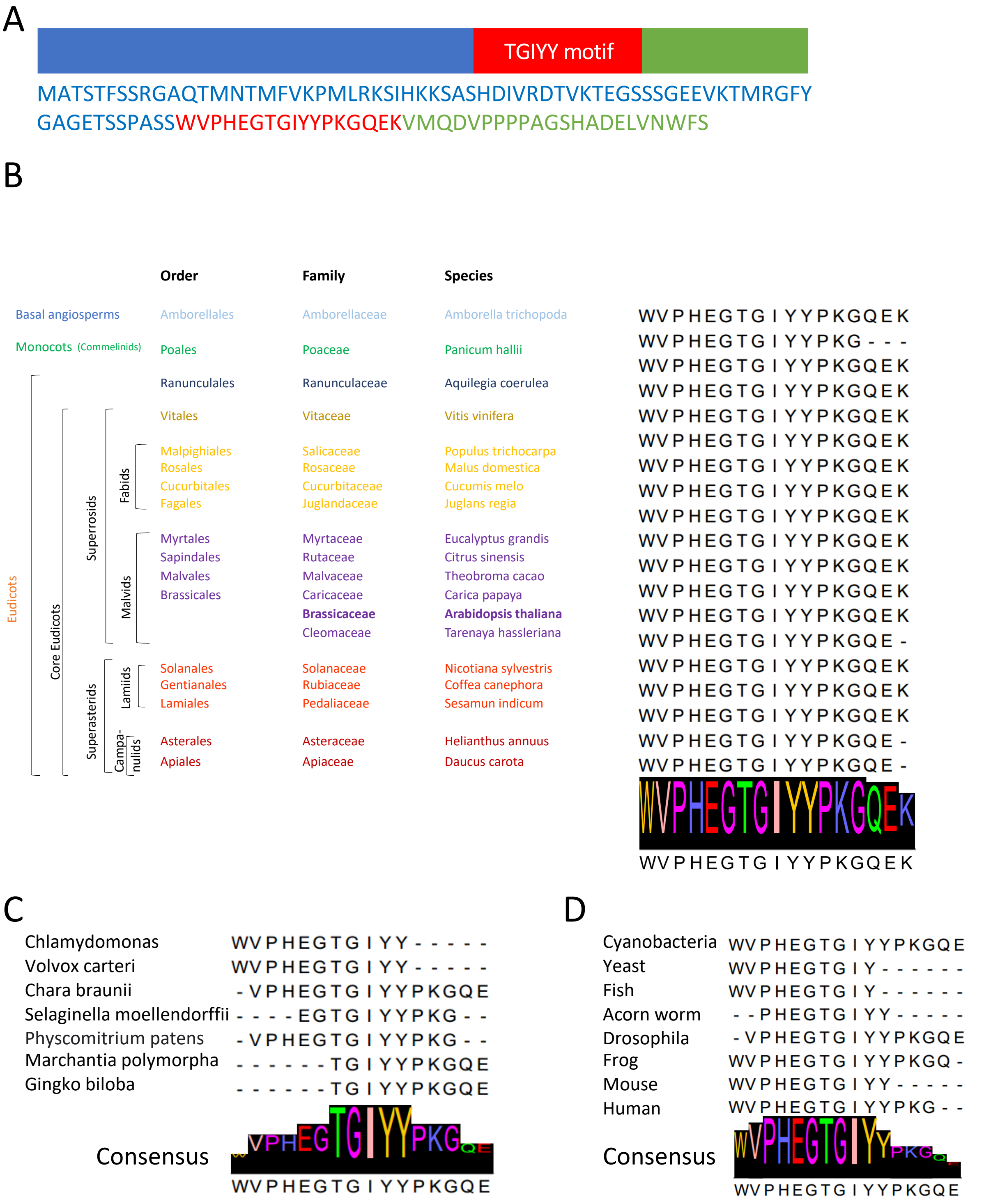

S3 Fig: Multiple sequence alignment of the TGIYY motifs from OLV orthologs of angiosperms and of TGIYY-containing proteins found in other organisms.

(A) Schematic representation of full length OLV and its amino acid sequence with N-terminus in blue, conserved TGIYY motif in red, C-terminus in green. **(B)** Multiple sequence alignment of the OLV TGIYY motifs of angiosperms. The ortholog with the highest maximum score similarity of each order is given. Multiple sequence alignments and consensus depicting TGIYY motifs from proteins found in **(C),** selected green algae and non-angiosperm land plants, and **(D)**, non-plant organisms.
