# Supplementary material for: The small iron-deficiency-induced protein OLIVIA and its relation to the bHLH transcription factor POPEYE
Source: PLoS One. 2024 Apr 16;19(4):e0295732. doi: 10.1371/journal.pone.0295732 (PMC11020826; doi:10.1371/journal.pone.0295732)
Supplement: S4 Fig — Subcellular localization in tobacco leaf epidermis cells. Proteins were N-terminally tagged to YFP. The OLV truncations are described in Fig 2A. Scale bar: 20 μm. (PDF) [file pone.0295732.s004.pdf]

S4 Fig

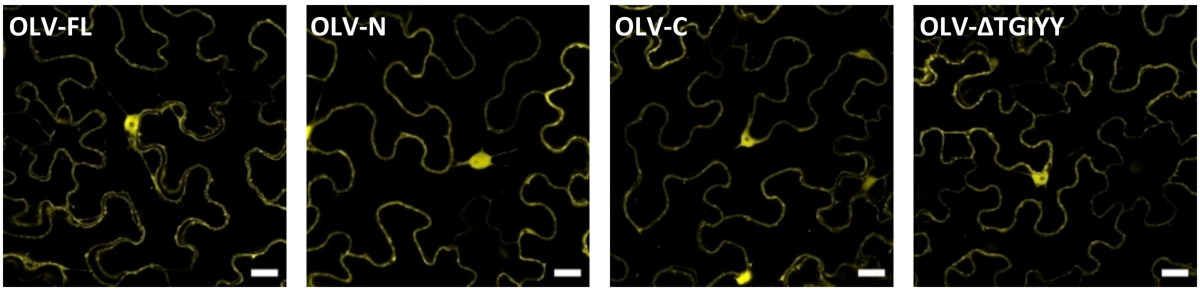

S4 Fig: Localization of various YFP-OLV fusions.

Subcelllar localization in tobacco leaf epidermis cells. Proteins were N-terminally tagged to YFP. The OLV truncations are described in Figure 2A. Scale bar: 20 µm.
