# Supplementary material for: The small iron-deficiency-induced protein OLIVIA and its relation to the bHLH transcription factor POPEYE
Source: PLoS One. 2024 Apr 16;19(4):e0295732. doi: 10.1371/journal.pone.0295732 (PMC11020826; doi:10.1371/journal.pone.0295732)
Supplement: S5 Fig — (A-D), Two OLV overexpression lines were investigated (OX7, OX11; pro2x35S promoter, triple hemagglutinine-tagged HA3-OLV). (A), Schematic overview of transgenic T-DNA construct, derived from pAlligator plasmid, not drawn to scale. (B) Anti-HA immunodetection of HA3-OLV protein in OX7 and OX11 grown under Fe-sufficient (+ Fe) and Fe-deficient (- Fe) conditions. Protein extracts were obtained from 10-d-old whole seedlings. PonceauS staining of the membrane served as loading control. Expected molecular weight of HA3-OLV: 14.85 kDa. Additional information in S1 File. (C, D) Gene expression analysis of OLV in (C) root and (D) shoot OX plants, WT and pye-1 mutant plants, as indicated in the figure. Plants were grown in the 9 + 3 d system with sufficient (+Fe) or deficient (-Fe) Fe supply for three days. (E-G), Two olv loss of function mutant lines, olv-3 and olv-7, were investigated. (E), Schematic representation of full length OLV and its amino acid sequence with N-terminus in blue, conserved TGIYY motif in red, C-terminus in green, comparison with two mutant OLV protein versions resulting from deletion/insertion following a genome editing procedure in olv-3 and olv-7. (F, G) Gene expression analysis of OLV in (F) root and (G) shoot of olv mutant, WT and pye-1 mutant plants, as indicated in the figure. Plants were grown as in (C, D). The data in (C, D, F, G) are depicted as mean ± standard deviations; n = 3. Different letters indicate statistically significant differences (one-way ANOVA and Tukey´s post-hoc test, p<0.05). Additional information in S3 File. (PDF) [file pone.0295732.s005.pdf]

S5 Fig

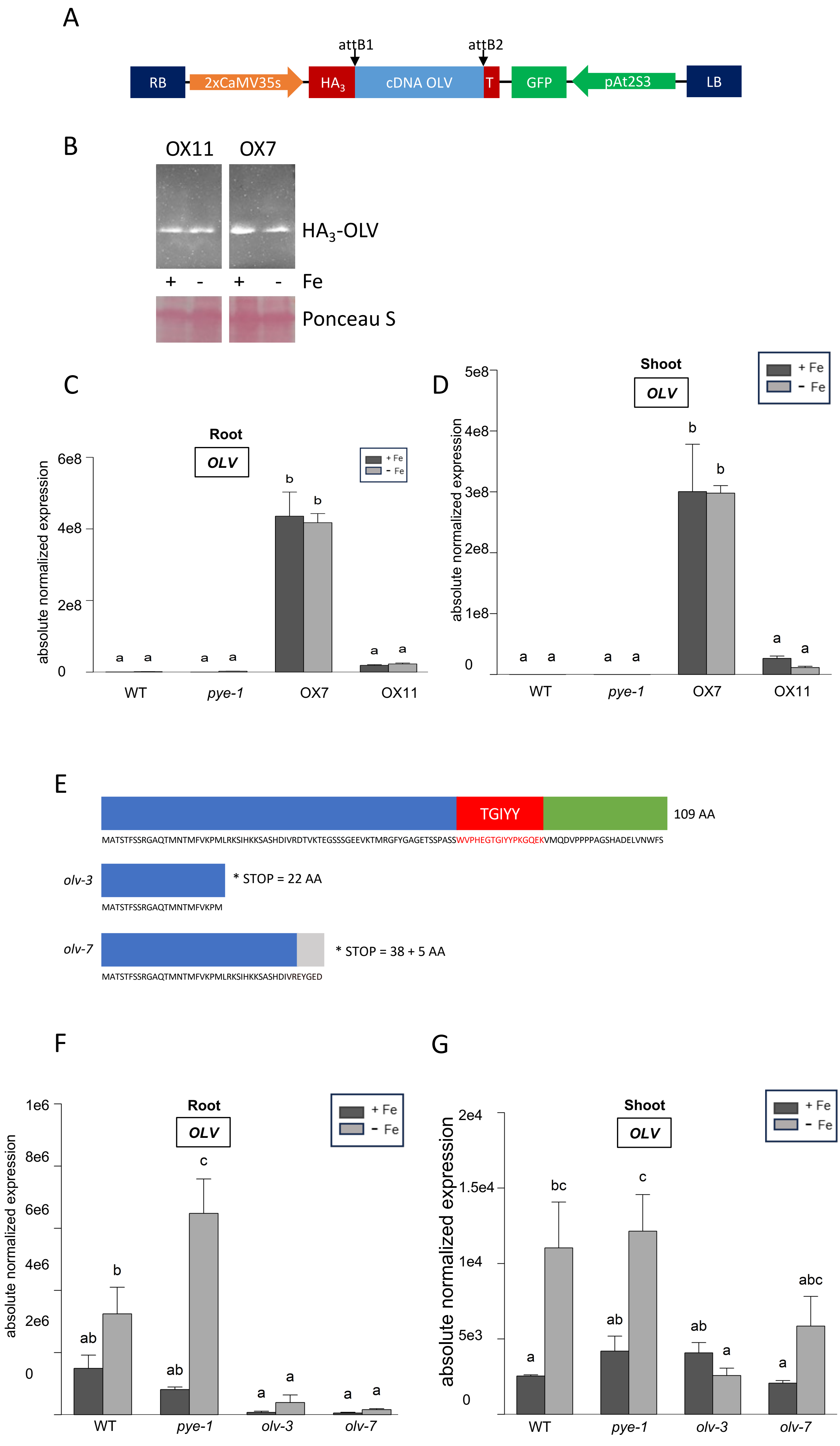
A
attB1
attB2
RB
2xCaMV35s
HA3
cDNA OLV
T
GFP
pAt2S3
LB
B
OX11
OX7
HA3-OLV
+ - + - Fe
Ponceau S
C
Root
OLV
+ Fe
- Fe
absolute normalized expression
6e8
4e8
2e8
0
a a a a b b a a
WT
pye-1
OX7
OX11
D
Shoot
OLV
+ Fe
- Fe
absolute normalized expression
5e8
4e8
3e8
2e8
1e8
0
a a a a b b a a
WT
pye-1
OX7
OX11
E
TGIYY
109 AA
MATSTFSSRGAQTMNTMFVKPMLRKSIHKKSASHDIVRDTVKTEGSSSGEEVKTMRGFYGAGETSSPASSWVPHEGTGIYYPKGQEKVMQDVPPPPAGSHADELVNWFS
olv-3
* STOP = 22 AA
MATSTFSSRGAQTMNTMFVKPM
olv-7
* STOP = 38 + 5 AA
MATSTFSSRGAQTMNTMFVKPMLRKSIHKKSASHDIVREYGED
F
Root
OLV
+ Fe
- Fe
absolute normalized expression
1e6
8e6
6e6
4e6
2e6
0
ab b ab c a a a a
WT
pye-1
olv-3
olv-7
G
Shoot
OLV
+ Fe
- Fe
absolute normalized expression
2e4
1.5e4
1e4
5e3
0
a bc ab c ab a a abc
WT
pye-1
olv-3
olv-7

S5 Fig. OLV overexpression and *olv* mutant lines.

(A-D), Two OLV overexpression lines were investigated (OX7, OX11; pro2x35S promoter, triple hemagglutinine-tagged HA_3-OLV). (A), Schematic overview of transgenic T-DNA construct, derived from pAlligator plasmid, not drawn to scale. (B) Anti-HA immunodetection of HA_3-OLV protein in OX7 and OX11 grown under Fe-sufficient (+ Fe) and Fe-deficient (- Fe) conditions. Protein extracts were obtained from 10-d-old whole seedlings. PonceauS staining of the membrane served as loading control. Expected molecular weight of HA_3-OLV: 14.85 kDa. Additional information in **S1 File**. (C, D) Gene expression analysis of *OLV* in (C) root and (D) shoot OX plants, WT and *pye-1* mutant plants, as indicated in the figure. Plants were grown in the 9 + 3 d system with sufficient (+Fe) or deficient (-Fe) Fe supply for three days. (E-G), Two *olv* loss of function mutant lines, *olv-3* and *olv-7*, were investigated. (E), Schematic representation of full length OLV and its amino acid sequence with N-terminus in blue, conserved TGIYY motif in red, C-terminus in green, comparison with two mutant OLV protein versions resulting from deletion/insertion following a genome editing procedure in *olv-3* and *olv-7*. (F, G) Gene expression analysis of *OLV* in (F) root and (G) shoot of *olv* mutant, WT and *pye-1* mutant plants, as indicated in the figure. Plants were grown as in (C, D). The data in (C, D, F, G) are depicted as mean ± standard deviations; n= 3. Different letters indicate statistically significant differences (one-way ANOVA and Tukey´s post-hoc test, $p<0.05$). Additional information in **S3 File**.
