# Supplementary material for: The small iron-deficiency-induced protein OLIVIA and its relation to the bHLH transcription factor POPEYE
Source: PLoS One. 2024 Apr 16;19(4):e0295732. doi: 10.1371/journal.pone.0295732 (PMC11020826; doi:10.1371/journal.pone.0295732)
Supplement: S6 Fig — Two OLV overexpression lines with pye-1 background were investigated (OX7, OX11; pro2x35S promoter, triple hemagglutinine-tagged HA3-OLV) and compared with wild type (WT) and pye-1. (A) Root lengths of 7-day-old seedlings grown in sufficient (+ Fe) or deficient (–Fe) Fe supply. (B-I) Gene expression analysis of PYE, FRO3, NAS4 and ZIF1 in root and shoot, as indicated in the figure. Plants were grown in the 9 + 3 d system with three-day + and–Fe treatments. The data are depicted as mean ± standard deviations; n = 3. Different letters indicate statistically significant differences (one-way ANOVA and Tukey´s post-hoc test, p<0.05). Green circles indicate significant differences to WT. Additional information in S2 and S3 Files. (PDF) [file pone.0295732.s006.pdf]

S6 Fig

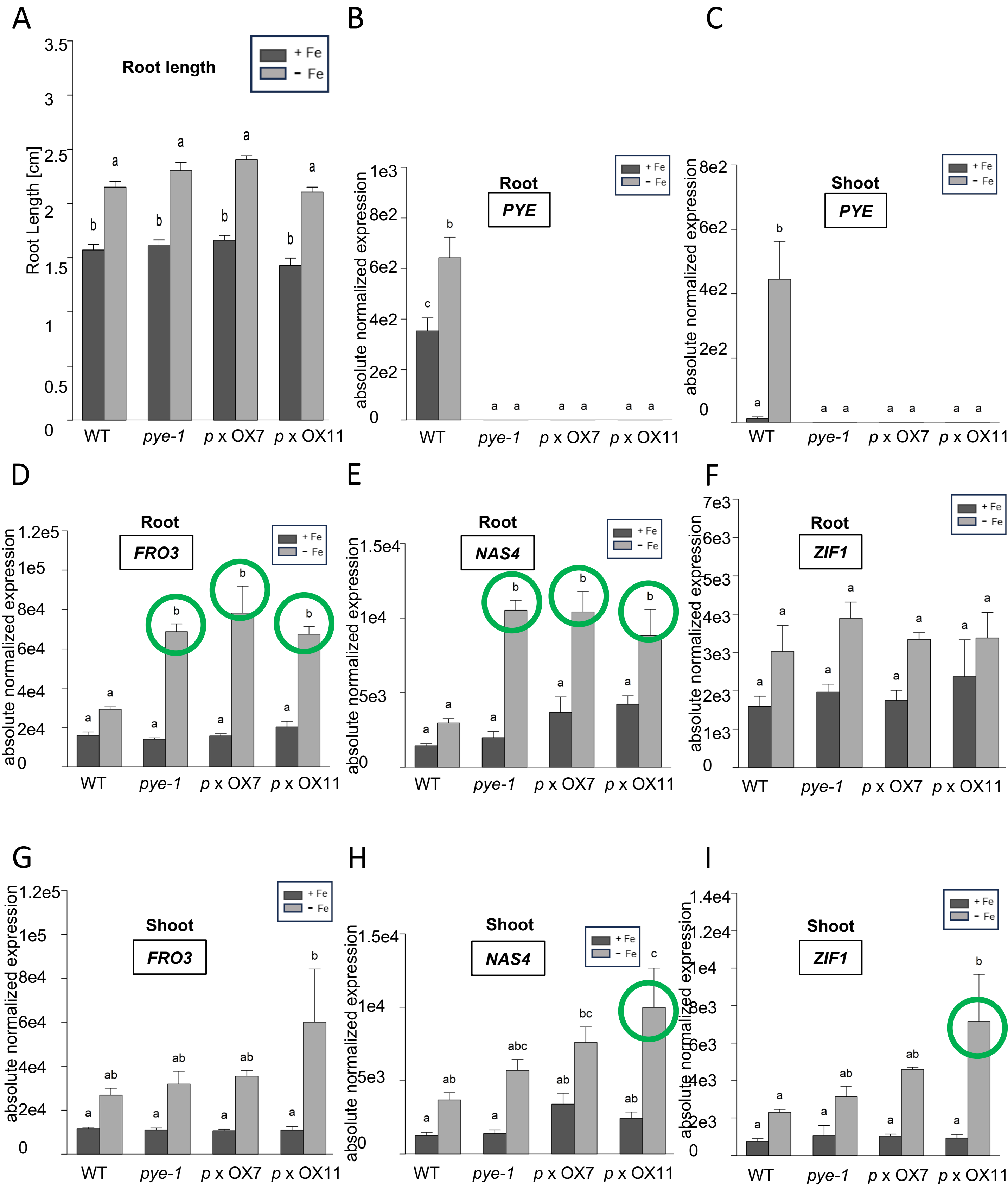
A
Root length
+ Fe
- Fe
Root Length [cm]
WT
pye-1
p x OX7
p x OX11
B
Root
PYE
C
Shoot
PYE
absolute normalized expression
D
Root
FRO3
E
Root
NAS4
F
Root
ZIF1
G
Shoot
FRO3
H
Shoot
NAS4
I
Shoot
ZIF1

S6 Fig: Overexpression of OLV in the background of *pye-1* did not alter the *pye-1* phenotype.

Two OLV overexpression lines with *pye-1* background were investigated (OX7, OX11; pro2x35S promoter, triple hemagglutinine-tagged HA_3-OLV) and compared with wild type (WT) and *pye-1.* (A) Root lengths of 7-day-old seedlings grown in sufficient (+ Fe) or deficient (– Fe) Fe supply. (B-I) Gene expression analysis of *PYE*, *FRO3*, *NAS4* and *ZIF1* in root and shoot, as indicated in the figure. Plants were grown in the 9 + 3 d system with three-day + and – Fe treatments. The data are depicted as mean ± standard deviations; n= 3. Different letters indicate statistically significant differences (one-way ANOVA and Tukey´s post-hoc test, $p<0.05$). Green circles indicate significant differences to WT. Additional information in **S2 and S3 Files**.
