# Supplementary material for: The small iron-deficiency-induced protein OLIVIA and its relation to the bHLH transcription factor POPEYE
Source: PLoS One. 2024 Apr 16;19(4):e0295732. doi: 10.1371/journal.pone.0295732 (PMC11020826; doi:10.1371/journal.pone.0295732)
Supplement: S1 Table — (DOCX) [file pone.0295732.s007.docx]

| **Supplemental Table S1: Primers used in this study.** | | | |
| --- | --- | --- | --- |
| **Primer Name** | **Sequence 5´ 🡪 3´** | **Application** | **Origin** |
| **Amplification of full-length or truncated CDS for Y2H, (co-) localization, BiFC, FRET and OX/ mutant lines** | | | |
| OLV_B1 fw | GGGGACAAGTTTGTACAAAAAAGCAGGCTTCATGGCGACTTCTACCTTCT | cloning of OLV-FL, OLV_1-55, 7320Δ | This study |
| OLVs_B2 rev | GGGGACCACTTTGTACAAGAAAGCTGGGTTCAAGAGAACCAATTAACGAG | cloning of OLV-FL, OLV_55-109, OLVΔ | This study |
| OLVns_B2 rev | GGGGACCACTTTGTACAAGAAAGCTGGGTCAGAGAACCAATTAACGAG | cloning of OLV-FL, OLV_55-109, OLVΔ | This study |
| OLV_B3 fw | GGGGACAACTTTGTATAATAAAGTTGTAATGGCGACTTCTACCTTCTC | cloning of OLV-FL, OLV_1-55 | This study |
| OLVs_B4 rev | GGGGACAACTTTGTATAGAAAAGTTGGGTGTCAAGAGAACCAATTAACGA | cloning of OLV-FL, OLV_55-109 | This study |
| OLVns_B4 rev | GGGGACAACTTTGTATAGAAAAGTTGGGTGAGAGAACCAATTAACGA | cloning of OLV-FL, OLV_55-109 | This study |
| OLV+165s_B2 rev | GGGGACCACTTTGTACAAGAAAGCTGGGTCTCACATCGTCTTCACCTCCT | cloning of OLV_1-55 | This study |
| OLV+165s_B4 rev | GGGGACAACTTTGTATAGAAAAGTTGGGTGTCACATCGTCTTCACCTCCT | cloning of OLV_1-55 | This study |
| OLV+165_B1 fw | GGGGACAAGTTTGTACAAAAAAGCAGGCTTCATGAGAGGCTTCTACGGCGCCGG | cloning of OLV_55-109 | This study |
| OLV+165_B3 fw | GGGGACAACTTTGTATAATAAAGTTGTAATGAGAGGCTTCTACGGCGCCGG | cloning of OLV_55-109 | This study |
| OLV+210_B1 fw | GGGGACAAGTTTGTACAAAAAAGCAGGCTTCATGTGGGTGCCACATGAAGGT | cloning of OLV motif | This study |
| OLV+264s_B2 rev | GGGGACCACTTTGTACAAGAAAGCTGGGTCTCATTTCTCTTGTCCCTTTGGATA | cloning of OLV motif | This study |
| OLV+210_B3 fw | GGGGACAACTTTGTATAATAAAGTTGTAATGTGGGTGCCACATGAAGGT | cloning of OLV motif | This study |
| OLV+264s_B4 rev | GGGGACAACTTTGTATAGAAAAGTTGGGTGTCATTTCTCTTGTCCCTTTGGATA | cloning of OLV motif | This study |
| OLVΔ fw | CGTCGTCGCCGGCGAGTAGTGTGATGCAAGATGTGCCTCC | cloning of OLVΔ | This study |
| OLVΔ rev | GGAGGCACATCTTGCATCACACTACTCGCCGGCGACGACG | cloning of OLVΔ | This study |
| OLV GW fw | GGGGACAAGTTTGTACAAAAAAGCAGGCTATGGCGACTTCTACCTTCTCTTCT | cloning of OLVΔ for FRET-APB | This study |
| OLV GW rv | GGGGACCACTTTGTACAAGAAAGCTGGGTCAGAGAACCAATTAACGAGCTCGTC | cloning of OLVΔ for FRET-APB | This study |
| OLV sgRNA_1 fw | ATTGCATGATATAGTGAGGGATA | cloning of *olv* | This study |
| OLV sgRNA_1 rev | AAACTATCCCTCACTATATCATG | cloning of olv | This study |
| OLV sgRNA_2 fw | ATTGTGAATTGATTTCCTAAGCAT | cloning of *olv* | This study |
| OLV sgRNA_2 rev | AAACATGCTTAGGAAATCAATTCA | cloning of *olv* | This study |
| FH41 | AAACGACGGCCAGTGCCAGAATTGGGCCCGACGTCG | cloning of *olv* | Hahn et al. 2017 |
| FH42 | TACTGACTCGTCGGGTACCAAGCTATGCATCCAACGCG | cloning of *olv* | Hahn et al. 2017 |
| FH254 | GCCCAATTCCAAGCTATGCATCCAACGCG | cloning of *olv* | Hahn et al. 2017 |
| FH255 | CATAGCTTGGAATTGGGCCCGACGTCG | cloning of *olv* | Hahn et al. 2017 |
| M13_CRISPR fw | GTAAAACGACGGCCAG | *olv* mutant line | Hahn et al. 2017 |
| M13_CRISPR rev | CAGGAAACAGCTATGAC | *olv* mutant line | Hahn et al. 2017 |
| PYE_B1 fw | GGGGACAAGTTTGTACAAAAAAGCAGGCTTCATGGTATCGAAAACTCCTTC | cloning of PYE | Lichtblau et al. 2021 |
| PYEs_B2 rev | GGGGACCACTTTGTACAAGAAAGCTGGGTCTCATTCACTGGCTTTCAGCC | cloning of PYE | Lichtblau et al. 2021 |
| PYEns_B2rev | GGGGACAAGTTTGTACAAAAAAGCAGGCTTCGAGAAAAGGATTCTAATTTAGGA | cloning of PYE | Lichtblau et al. 2021 |
| PYE_B3 fw | GGGGACAACTTTGTATAATAAAGTTGTAATGGTATCGAAAACTCCTTC | cloning of PYE | Lichtblau et al. 2021 |
| PYEs_B4 rev | GGGGACAACTTTGTATAGAAAAGTTGGGTGTCATTCACTGGCTTTCAGCC | cloning of PYE | Lichtblau et al. 2021 |
| PYEns_B4 rev | GGGGACAACTTTGTATAGAAAAGTTGGGTGTTCACTGGCTTTCAGCC | cloning of PYE | Lichtblau et al. 2021 |
| PYE+361_attB1 fw | GGGGACAAGTTTGTACAAAAAAGCAGGCTTCATGAACGAGATTGAAGCTAG | Cloning of PYE-C deletion construct | This study |
| PYE_attB1 fw +81bp | GGGGACAAGTTTGTACAAAAAAGCAGGCTTCATGAAAAGGATCAACAAGGCC | Cloning of PYE-bHLH deletion construct | This study |
| PYE_attB2 rev +231bp | GGGGACCACTTTGTACAAGAAAGCTGGGTCTCAAAACACGTCCTTCAAGAA | Cloning of PYE-bHLH deletion construct | This study |
| PYE+360_attB2 rev | GGGGACCACTTTGTACAAGAAAGCTGGGTCTCATTGTAGTTTCGAAATCT | Cloning of PYE-N deletion construct | This study |
| PYE_ΔbHLH fw | GTAGAAAAGGTAAAGTACCGGGTCAAATTGAGTCTCTTAG | Cloning of PYE_ΔbHLH deletion construct | This study |
| PYE_ΔbHLH rev | CTAAGAGACTCAATTTGACCCGGTACTTTACCTTTTCTAC | Cloning of PYE_ΔbHLH deletion construct | This study |
| PYE_ΔEAR fw | AGAGCGAATCAGTCGAAACCTGCACCCGAGTACCATCATCA | Cloning of PYE_ΔEAR deletion construct | This study |
| PYE_ΔEAR rev | TGATGATGGTACTCGGGTGCAGGTTTCGACTGATTCGCTC | Cloning of PYE_ΔEAR deletion construct | This study |
| ILR3_B1 fw | GGGGACAAGTTTGTACAAAAAAGCAGGCTTCATGGTGTCACCCGAAAACG | colony PCR ILR3 | Lichtblau et al. 2021 |
| ILR3_B2 rev | GGGGACCACTTTGTACAAGAAAGCTGGGTCTTAAGCAACAGGAGGACGAAG | colony PCR ILR3 | Lichtblau et al. 2021 |
| bHLH39_B1 fw | GGGGACAAGTTTGTACAAAAAAGCAGGCTTCATGTGTGCATTAGTACCTC | colony PCR bHLH39 | Lichtblau et al. 2021 |
| bHLH39_B2 rev | GGGGACCACTTTGTACAAGAAAGCTGGGTCTCATATATATGAGTTTCCAC | colony PCR bHLH39 | Lichtblau et al. 2021 |
| **Amplification of promotor sequences for promotor-GUS lines** | | | |
| proOLV_-988_B1 fw | GGGGACAAGTTTGTACAAAAAAGCAGGCTTCGAGAAAAGGATTCTAATTTAGGA | cloning of OLV promotor | This study |
| ProOLV_-988_B2 rev | GGGGACCACTTTGTACAAGAAAGCTGGGTCGTAAATCTCTATGGTCTATTG | cloning of OLV promotor | This study |
| proPYE_-1120_B1 fw | GGGGACAAGTTTGTACAAAAAAGCAGGCTTCACCGCAAAACTATATATAGTATTT | cloning of PYE promotor | Lichtblau et al. 2021 |
| proPYE_-1120_B2 rev | GGGGACCACTTTGTACAAGAAAGCTGGGTCCTTTGCTTTTATTACAGAACAAGA | cloning of PYE promotor | Lichtblau et al. 2021 |
| **Genotyping primer** | | | |
| 35S fw | ATCCCACTATCCTTCGCAAGACCC | HA-ox lines | Institute of Botany, HHU Düsseldorf |
| pGWB3_seq fw | TACACTTTATGCTTCCGGCTC | Gus lines | This study |
| pGWB3_seq rev | CTTTCCCACCAACGCTGATC | Gus lines | This study |
| OLV genotyping_F | GGGCACAAACCATGAACACC | Genome-edited lines | This study |
| OLV genotyping_R | GAGTCTTCGACGAGATCGAGATG | Genome-edited lines | This study |
| OLV sequencing_F | ATGGCGACTTCTACCTTCT | Genome-edited lines | This study |
| OLV sequencing_R | GAGTCTTCGACGAGATCGAGATG | Genome-edited lines | This study |
| PYE WT_F | TTCAAGACCTCATTCACTGGC | WT/*pye1-1* | This study |
| PYE WT_R | ATCGTCTGATGAAGCAAATGC | WT/*pye1-1* | This study |
| *pye-1*_F | ATTTTGCCGATTTCGGAAC | WT/*pye1-1* | This study |
| *pye-1*_R | ATCGTCTGATGAAGCAAATGC | WT/*pye1-1* | This study |
| **RT-qPCR primer** | | | |
| OLV_stn fw | GGGCACAAACCATGAACACC | OLV_RT-qPCR mass standard | This study |
| OLV_stn rev | GAGTCTTCGACGAGATCGAGATG | OLV_RT-qPCR mass standard | This study |
| OLV_qPCR fw | GGGCACAAACCATGAACACC | OLV_RT-qPCR | This study |
| OLV_qPCR rev | ACTATATCATGCGAGGCACTCTTC | OLV_RT-qPCR | This study |
| PYE_stn fw | ACCGAAAAGGATCAACAAGG | PYE_RT-qPCR mass standard | Lichtblau et al. 2021 |
| PYE_stn rev | CCATCAAGGCCATAACTTCC | PYE_RT-qPCR mass standard | Lichtblau et al. 2021 |
| PYE_qPCR fw | GTTCCCAGGACTTCCCATTT | PYE_RT-qPCR | Lichtblau et al. 2021 |
| PYE_qPCR rev | GTGTCTGGGGATCAGGTTGT | PYE_RT-qPCR | Lichtblau et al. 2021 |
| NAS4_stn fw | CACTCTCTTCAAGCAGCTCGT | NAS4_RT-qPCR mass standard | Lichtblau and Schwarz et al. 2020 |
| NAS4_stn rev | CTGTAGCAAAAACAGCCAACA | FAS4_RT-qPCR mass standard | Lichtblau and Schwarz et al. 2020 |
| AtNAS4-RT810-5´ | TGTAATCTCAAGGAAGCTAGGTG | NAS4_RT-qPCR | Klatte et al., 2009 |
| AtNAS4-RT947-3´ | GCGAACTCCTCGATAATGC | NAS4_RT-qPCR | Institute of Botany, HHU Düsseldorf |
| FRO3_stn fw | AATCAGATCGACCACCTTGC | FRO3_RT-qPCR mass standard | Lichtblau and Schwarz et al. 2020 |
| FRO3_stn rev | TTCTTTTGGTGAGAAGATTTTGG | FRO3_RT-qPCR mass standard | Lichtblau and Schwarz et al. 2020 |
| FRO3_qPCR fw | ATCGACCACCTTGCTGTTTC | FRO3_RT-qPCR | Lichtblau and Schwarz et al. 2020 |
| FRO3_qPCR rev | TTATCCCACTGCCTCCACTC | FRO3_RT-qPCR | Lichtblau and Schwarz et al. 2020 |
| ZIF1_stn fw | AAGGCTTCTCAGTCTCTCTTG | ZIF1_RT-qPCR mass standard | This study |
| ZIF1_stn rev | TAACGGTTCAAGTAAGTTCCTCTC | ZIF1_RT-qPCR mass standard | This study |
| ZIF1_qPCR fw | TTGGCTGAGAAACTGCTAGG | ZIF1_RT-qPCR | This study |
| ZIF1_qPCR rev | CTTAGACTGAGACCTGACAAGC | ZIF1_RT-qPCR | This study |
| OPT3_stn fw | TCGGTTATATCCTGCCTG | OPT3_RT-qPCR mass standard | This study |
| OPT3_stn rev | GACAGATGTCTCAATAGCTC | OPT3_RT-qPCR mass standard | This study |
| OPT3_qPCR fw | TGATAGGACCAAGACGGCTC | OPT3_RT-qPCR | This study |
| OPT3_qPCR rev | GCAAAGCCGTAGGAGATAACTG | OPT3_RT-qPCR | This study |
| STD-EF1Balpha2-5´ | GCTGCTAAGAAGGACACCAAG | EF1Balpha (genomic) RT-qPCR mass standard | (Bauer, 2016) |
| STD-EF1Baplha2-3´ | TGTTCTGTCCCTACGGATCC | EF1Balpha (genomic) RT-qPCR mass standard | (Bauer 2016) |
| EFc-5´ | TATGGGATCAAGAAACTCACAAT | EF1Balpha RT-qPCR | (Bauer, 2016) |
| EFc-3´ | CTGGATGTACTCGTTGTTAGGC | EF1Balpha RT-qPCR | (Wang et al., 2007) |
| At-EF-gen-5´ | TCCGAACAATACCAGAACTAC | EF1Balpha (genomic) RT-qPCR | (Wang et al. 2007) |
| At-EF-gen-3´ | CCGGGACATATGGAGGTAAG | EF1Balpha (genomic) RT-qPCR | (Wang et al., 2007) |
| Fw = forward, rev = reverse, s = stop codon, ns = no stop codon, FL = full length, Δ = delta conserved motif, B1/B2/B3/B4 = (attb) gateway attachment sites | | | |
