# Supplementary material for: The small iron-deficiency-induced protein OLIVIA and its relation to the bHLH transcription factor POPEYE
Source: PLoS One. 2024 Apr 16;19(4):e0295732. doi: 10.1371/journal.pone.0295732 (PMC11020826; doi:10.1371/journal.pone.0295732)
Supplement: S1 File — (PDF) [file pone.0295732.s008.pdf]

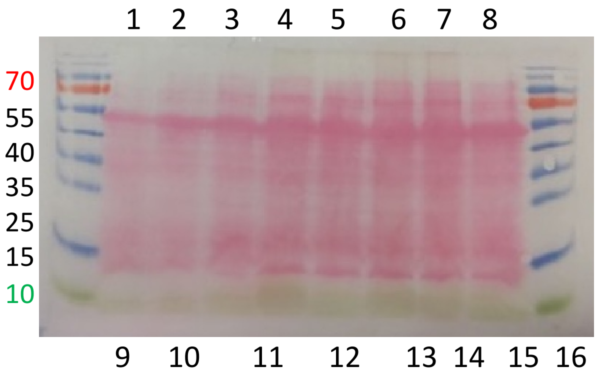

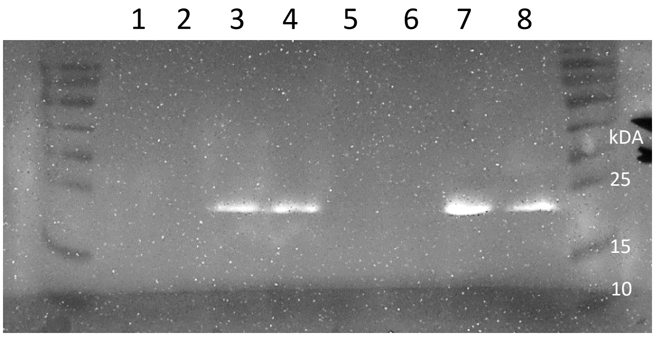

Samples

1. Col-0 –Fe
2. Col-0 + Fe
3. OX11 –Fe
4. OX11 +Fe
5. OX –Fe → no Signal
6. OX + Fe → no Signal
7. OX7 –Fe
8. OX7 +Fe

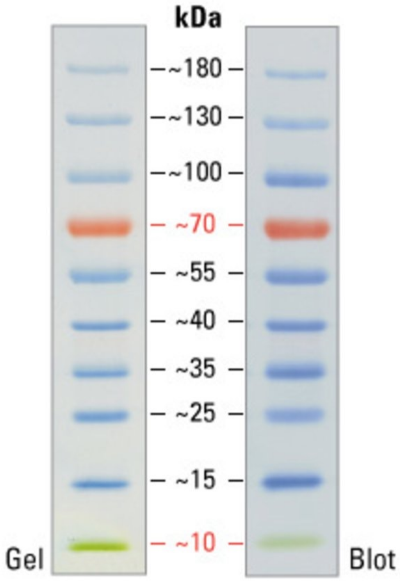

Original immunoblot, supporting Fig S5B
