# Supplementary material for: The small iron-deficiency-induced protein OLIVIA and its relation to the bHLH transcription factor POPEYE
Source: PLoS One. 2024 Apr 16;19(4):e0295732. doi: 10.1371/journal.pone.0295732 (PMC11020826; doi:10.1371/journal.pone.0295732)
Supplement: Attachment — Submitted filename: Rebuttal Letter.pdf [file pone.0295732.s017.pdf]

Rebuttal Letter

Dear Editor and reviewers,

We thank you for appreciating our manuscript and very thoroughly studying it to make valuable comments. Please find below our answers to your comments and we hope that we could address them appropriately. We made minor improvements and corrections in the text wherever appropriate and according to reviewer comments.

Thank you for your time and looking forward to your response,

Sincerely

Petra Bauer

1. When submitting your revision, we need you to address these additional requirements.

Please ensure that your manuscript meets PLOS ONE's style requirements, including those for file naming. The PLOS ONE style templates can be found at https://journals.plos.org/plosone/s/file?id=wjVg/PLOSOne_formatting_sample_main_body.pdf and https://journals.plos.org/plosone/s/file?id=ba62/PLOSOne_formatting_sample_title_authors_affiliations.pdf.

Our response: To the best of our knowledge, our manuscript now meets the PLoS One style requirements.

2. Note from Emily Chenette, Editor in Chief of PLOS ONE, and Iain Hrynaszkiewicz, Director of Open Research Solutions at PLOS: Did you know that depositing data in a repository is associated with up to a 25% citation advantage (https://doi.org/10.1371/journal.pone.0230416)? If you've not already done so, consider depositing your raw data in a repository to ensure your work is read, appreciated and cited by the largest possible audience. You'll also earn an Accessible Data icon on your published paper if you deposit your data in any participating repository (https://plos.org/open-science/open-data/#accessible-data).

Our response: We deposited the FRET-APB imaging data in a repository at https://www.ebi.ac.uk/biostudies/bioimages/studies/S-BIAD1028 with the accession number S-BIAD1028.

3. Thank you for stating the following financial disclosure:
[This work was funded by Deutsche Forschungsgemeinschaft (DFG, German Research Foundation) under GRK F020512056 (NEXTplant) and Germany´s Excellence Strategy – EXC-2048/1 – project ID 390686111. Funding for instrumentation: Zeiss LSM780 + 4-channel FLIM extension (Picoquant): DFG- INST 208/551-1 FUGG.].
Please state what role the funders took in the study. If the funders had no role, please state: ""The funders had no role in study design, data collection and analysis, decision to publish, or preparation of the manuscript.""
If this statement is not correct you must amend it as needed.

Please include this amended Role of Funder statement in your cover letter; we will change the online submission form on your behalf.

Our response: This work was funded by Deutsche Forschungsgemeinschaft (DFG, German Research Foundation) under GRK F020512056 (NEXTplant) and Germany´s Excellence Strategy – EXC-2048/1 – project ID 390686111. Funding for instrumentation: Zeiss LSM780 + 4-channel FLIM extension (Picoquant): DFG- INST 208/551-1 FUGG. We state that ""The funders had no role in study design, data collection and analysis, decision to publish, or preparation of the manuscript.""
The information about funding must appear in the manuscript. Please add it where appropriate.

5. In the online submission form, you indicated that [Data will be published. Seeds and original data can be made available upon request.].
All PLOS journals now require all data underlying the findings described in their manuscript to be freely available to other researchers, either 1. In a public repository, 2. Within the manuscript itself, or 3. Uploaded as supplementary information.
This policy applies to all data except where public deposition would breach compliance with the protocol approved by your research ethics board. If your data cannot be made publicly available for ethical or legal reasons (e.g., public availability would compromise patient privacy), please explain your reasons on resubmission and your exemption request will be escalated for approval.

Our response: We have uploaded the following files:
Supporting Information file. Original blot images.

Supporting Information file. Fe contents young leaves_Fig 7E. Original data.

Supporting Information file. Fe contents old leaves_Fig 7F. Original data.

Supporting Information file. Zn contents young leaves_Fig 7G. Original data.

Supporting Information file. Zn contents old leaves_Fig 7H. Original data.

Supporting Information file. Rosette size_Fig 7C. Original data.

Supporting Information file. Rosette size_Fig 7D. Original data.

Supporting Information file. Root lengths 7-day seedlings_Figs 5A+6A+S6A. Original data.

Supporting Information file. Gene expression values-Figs 5B-I and 6B-I and S5C, D, F, G and S6B-I. Original data.

6. PLOS ONE now requires that authors provide the original uncropped and unadjusted images underlying all blot or gel results reported in a submission's figures or Supporting Information files. This policy and the journal's other requirements for blot/gel reporting and figure preparation are described in detail at https://journals.plos.org/plosone/s/figures#loc-blot-and-gel-reporting-requirements and https://journals.plos.org/plosone/s/figures#loc-preparing-figures-from-image-files. When you submit your revised manuscript, please ensure that your figures adhere fully to these guidelines and provide the original underlying images for all blot or gel data reported in your submission. See the following link for instructions on providing the original image data: https://journals.plos.org/plosone/s/figures#loc-original-images-for-blots-and-gels.

In your cover letter, please note whether your blot/gel image data are in Supporting Information or posted at a public data repository, provide the repository URL if relevant, and provide specific details as to which raw blot/gel images, if any, are not available. Email us at plosone@plos.org if you have any questions.

Our response: We have provided the original blots as supporting information file. Original FRET-APB imaging data sets are available at https://www.ebi.ac.uk/biostudies/bioimages/studies/S-BIAD1028 with the accession number S-BIAD1028 as stated in the Mat Meth section of the revised manuscript.

7. We note that Figure(s) 1A, 1C, 2B, 2D, 3B, 4A,B, C, D, E. 7A, 7B and S4 in your submission contain copyrighted images. All PLOS content is published under the Creative Commons Attribution License (CC BY 4.0), which means that the manuscript, images, and Supporting Information files will be freely available online, and any third party is permitted to access, download, copy, distribute, and use these materials in any way, even commercially, with proper attribution. For more information, see our copyright guidelines: http://journals.plos.org/plosone/s/licenses-and-copyright.

We require you to either (1) present written permission from the copyright holder to publish these figures specifically under the CC BY 4.0 license, or (2) remove the figures from your submission:

a. You may seek permission from the original copyright holder of Figure(s) 1A, 1C, 2B, 2D, 3B, 4A,B, C, D, E. 7A, 7B and S4 to publish the content specifically under the CC BY 4.0 license.

We recommend that you contact the original copyright holder with the Content Permission Form (http://journals.plos.org/plosone/s/file?id=7c09/content-permission-form.pdf) and the following text:
“I request permission for the open-access journal PLOS ONE to publish XXX under the Creative Commons Attribution License (CCAL) CC BY 4.0 (http://creativecommons.org/licenses/by/4.0/). Please be aware that this license allows unrestricted use and distribution, even commercially, by third parties. Please reply and provide explicit written permission to publish XXX under a CC BY license and complete the attached form.”

Our response: In our view our figures of the preprint on biorxiv are available under CC BY 4.0 licence. Please let us know if that is not sufficient. bioRxiv preprint doi: https://doi.org/10.1101/2023.12.05.570225; this version posted December 6, 2023. The copyright holder for this preprint (which was not certified by peer review) is the author/funder, who has granted bioRxiv a license to display the preprint in perpetuity. It is made available under aCC-BY 4.0 International license. If biorxiv is not the publication place that you are referring to, please kindly let us know which publication you mean.

Please upload the completed Content Permission Form or other proof of granted permissions as an ""Other"" file with your submission.

In the figure caption of the copyrighted figure, please include the following text: “Reprinted from [ref] under a CC BY license, with permission from [name of publisher], original copyright [original copyright year].”
Our response: We do not think, there are copy-righted figures. Please instruct.

b. If you are unable to obtain permission from the original copyright holder to publish these figures under the CC BY 4.0 license or if the copyright holder’s requirements are incompatible with the CC BY 4.0 license, please either i) remove the figure or ii) supply a replacement figure that complies with the CC BY 4.0 license. Please check copyright information on all replacement figures and update the

figure caption with source information. If applicable, please specify in the figure caption text when a figure is similar but not identical to the original image and is therefore for illustrative purposes only.

Our response: The copyright holder for this preprint is the author/funder, who has granted bioRxiv a license to display the preprint in perpetuity. It is made available under a CC-BY 4.0 International license.

Please let us know if there is anything unclear with it.

8. Please include captions for your Supporting Information files at the end of your manuscript, and update any in-text citations to match accordingly. Please see our Supporting Information guidelines for more information: http://journals.plos.org/plosone/s/supporting-information.

We added the legends to supplemental data files at the end of the manuscript.

Comments to the Author

Reviewer #1:

Figures:
Comment 1.
In Figure 4D and E, proOLV::GUS activity was induced by -Fe treatment in root differentiation zone and hair. Did the authors test the transcript level of OLV by qPCR at same developmental stage to show that induction is relevant? Seems the expression of OLV is not increased by -Fe in WT (see Fig S5 C).

Our response: OLV is co-expressed with iron deficiency-induced genes. In Fig. S5C, the focus is on strong expression in the OLV OX lines, and the representation does not depict differences in OLV expression in the wild type as it is represented along with the overexpressors. In Fig. S5F, there is an induction of OLV expression upon -Fe versus +Fe which is significant in pye. In Fig. S5G, there is a significant induction of OLV in WT and pye.

Comment 2.
In Figure 4F and G shows, proPYE::GUS mainly detected in root tissue only. Why proPYE::GUS activity did not induce in leaves (or shoot) although PYE expression was highly induced both root and shoot by -Fe in Fig 5C (in WT). How do you explain?

Our response: OLV is expressed primarily in roots, and we have been interested in the comparison with PYE expression in roots. The utilized GUS assay procedure works well for root staining. For cotyledon staining, infiltration, cuttings or longer incubation times may have to be used to make GUS substrate penetrate. Please also note that GUS staining is not quantitative. We added: The lines were grown for six days under sufficient (+Fe) or deficient (–Fe) Fe supply and analyzed for promoter-GUS activity in roots (**Figure 4D, E, F, G**).

Comment 3.
Different letters need improving in Fig5 and Fig6 diagrams. For instance, same letter indicated for all samples in Figure 6F. Seems there are some significant differences between treatment. Similar other errors: Fig5C “ab”, Fig5C “abc”, Fig6B “ab”, Fig6C “ab”.

Our response: We have checked. The representation is correct.

Comment 4.
Y axis needs improving in Fig 5A and Fig 6A. Please double check.

Our response: Thank you, we corrected it.

Comment 5.
Authors used two independent OLV overexpression lines (OX7 and OX11) in all experiments. Expression of OLV was not increased in OX11 (see Fig S5 C and D).

Our response: Thank you. There is moderate overexpression. The difference to wild type has not been found significant. Fig. S5B shows that there is HA3-OLV protein. We now write: At first, we assessed a possible impact of OLV overexpression (meaning expression behind the double cauliflower mosaic virus 35S promoter).

Methods:
Comment 6.
What type of pye-1 mutant was used. Describe it in Plant material section.

Our response: Thank you, we added it.

Comment 7.
Please, complete the description of protocol. What kind of secondary antibody was used.

Our response: anti-HA high-affinity monoclonal rat antibody - conjugated with horseradish peroxidase (clone 3F10; Roche), a secondary antibody is not needed.

Typo errors:
Typographical error in “rot” instead of root (Page9)
Typographical error in “bHLHIVc” (Page5)
Typographical error in “wasused” (Page15)
Typographical error in Fig S4 legend “Subcelllar”.

Our response: Thank you, was done.

Reviewer #2: Article review
1. EVALUATION OF THE PAPER MANUSCRIPT
Title of the Manuscript:
"The small iron-deficiency-induced protein OLIVIA and its relation to the bHLH transcription factor POPEYE"
Manuscript number: PONE-D-23-39597

The article PONE-D-23-39597 has comprehensively demonstrated that Fe deficiency-induced bHLH transcription factor (POPEYE) can bind to the small protein OLIVIA (OLV), which is induced by Fe scarcity. The article PONE-D-23-39597 is slightly suited to the PloS One as per its aims and scope. In general, the manuscript is written in good logical style with a suitable introduction, interesting and

reasonable Results and Discussion sections. Therefore, the manuscript can be conditionally accepted. However, to improve the quality of the article, some minor points should be noted:
- Could the authors re-analyze RNA-Seq datasets to see how the OLV gene is expressed in different organs/tissues during growth and development? If so, please write several sentences describing the expression level of the OLV gene.

Our response: Thank you, we added: According to available transcriptome data under control conditions, *OLV* is expressed during germination and in the root epidermis (Klepikova et al., 2016; Ryu et al., 2019).

- The authors should include more information about the OLV in the conclusion section. Also, if possible, please simplify and combine the perspectives into a single paragraph.

Our response: Thank you, we shortened the conclusions/perspectives paragraph: OLV is a small protein interactor of PYE that may interact via a conserved previously non-characterized TGIYY motif in the Fe deficiency response. OLV had a mild effect on plant growth and Fe deficiency responses involving *NAS4*. Since *NAS4* is specifically targeted by other TFs of the Fe deficiency response, OLV may control a particular sub-function of PYE in controlling *NAS4*. OLV is primarily expressed during germination and in in roots where it is induced by Fe deficiency. Future studies can address the functional and structural relevance of the TGIYY motif for formation of the protein complex with PYE. Protein interaction can be further studied in plant roots, e.g. by co-expressing fusion proteins of OLV and PYE expressed from their respective promoters.

- All abbreviations should be applied after the first occurrence, such as transcription factor (pages 5 and 11).

Our response: Thank you, was done.

- List of references should be exactly followed the PloS One's style.

Our response: Is now the correct style.

- A minor issue is that the reviewer is unable to access the link to jmicrovision. If possible, offer a new link.

Our response: Thank you, we corrected it.
