# Supplementary material for: The small iron-deficiency-induced protein OLIVIA and its relation to the bHLH transcription factor POPEYE
Source: PLoS One. 2024 Apr 16;19(4):e0295732. doi: 10.1371/journal.pone.0295732 (PMC11020826; doi:10.1371/journal.pone.0295732)
Supplement: Attachment — Submitted filename: Rebuttal Letter 3_2024.pdf [file pone.0295732.s018.pdf]

Rebuttal Letter

Dear Editor and reviewers,

We thank you for appreciating our revised manuscript and for very thoroughly studying the revision to make valuable comments. Please find below our answers to your comments. All corrections were made and we hope that we could address them appropriately.

Thank you for your time and looking forward to your response,
Hopefully our manuscript is now ready for acceptance,

Sincerely

Petra Bauer

Reviewrs' comments:

Reviewer's Responses to Questions

Comments to the Author

1. If the authors have adequately addressed your comments raised in a previous round of review and you feel that this manuscript is now acceptable for publication, you may indicate that here to bypass the “Comments to the Author” section, enter your conflict of interest statement in the “Confidential to Editor” section, and submit your "Accept" recommendation.

Reviewer #1: (No Response)

Reviewer #2: All comments have been addressed

---

2. Is the manuscript technically sound, and do the data support the conclusions?

The manuscript must describe a technically sound piece of scientific research with data that supports the conclusions. Experiments must have been conducted rigorously, with appropriate controls, replication, and sample sizes. The conclusions must be drawn appropriately based on the data presented.

Reviewer #1: Yes

Reviewer #2: Yes

---

3. Has the statistical analysis been performed appropriately and rigorously?

Reviewer #1: Yes

Reviewer #2: Yes

---

4. Have the authors made all data underlying the findings in their manuscript fully available?

The PLOS Data policy requires authors to make all data underlying the findings described in their manuscript fully

available without restriction, with rare exception (please refer to the Data Availability Statement in the manuscript PDF file). The data should be provided as part of the manuscript or its supporting information, or deposited to a public repository. For example, in addition to summary statistics, the data points behind means, medians and variance measures should be available. If there are restrictions on publicly sharing data—e.g. participant privacy or use of data from a third party—those must be specified.

Reviewer #1: Yes

Reviewer #2: Yes

---

5. Is the manuscript presented in an intelligible fashion and written in standard English?

PLOS ONE does not copyedit accepted manuscripts, so the language in submitted articles must be clear, correct, and unambiguous. Any typographical or grammatical errors should be corrected at revision, so please note any specific errors here.

Reviewer #1: Yes

Reviewer #2: Yes

---

6. Review Comments to the Author

Please use the space provided to explain your answers to the questions above. You may also include additional comments for the author, including concerns about dual publication, research ethics, or publication ethics. (Please upload your review as an attachment if it exceeds 20,000 characters)

Reviewer #1: Comment1:
In page 5, you mentioned “OLV also affects biomass”. Indeed, olv mutant showed smaller phenotype compared to WT (supported by rosette size data), but you do not have fresh weight value. Please, think on that, or just remove “biomass”.

We exchanged “biomass” against “rosette size”.

Typo errors:
Typographical error in “BHLH” (Page5). Usually, we use bHLH.

bHLH designates a protein; *BHLH* designates DNA/RNA/gene. We checked for correctness throughout the text.

Typographical error in “Sesanum indicum” (Page 7).

corrected

Typographical errors in FigS2. Please, double check the name of genus: “Actinida chinensis “, “Sesamum idicum”, “Viits vinifera”.

corrected

Typographical errors in FigS3. Please, double check “Sesamun indicum “, “Gingko biloba”.

corrected

Typographical error in FigS4 “Subcelllar”.

corrected

Our response: The authors thank reviewer 1 for suggestions. We corrected all of the spelling mistakes.

Reviewer #2: All comments have been addressed. The reviewer believes that this manuscript version meets the requirements for publication in PloS ONE.

Our response: The authors thank reviewer 2 for assessment.

---

7. PLOS authors have the option to publish the peer review history of their article (what does this mean?). If published, this will include your full peer review and any attached files.

If you choose “no”, your identity will remain anonymous but your review may still be made public.

Do you want your identity to be public for this peer review? For information about this choice, including consent withdrawal, please see our Privacy Policy.

Reviewer #1: **Yes:** Shapulatov Umidjon

Reviewer #2: No
